# Ectopic Papillary Thyroid Cancer: About a Case

**DOI:** 10.7759/cureus.72072

**Published:** 2024-10-21

**Authors:** Jaime Enrique Hernández-Utrera, Luis Fernando Domínguez-Valdez, Alejandra Lara-Mejía, José Roberto Juárez-Díaz, Abraham Edgar Gracia-Ramos, Antonio Segovia-Palomo, Héctor A Rodríguez-Rubio

**Affiliations:** 1 Internal Medicine, Specialties Hospital "Dr. Antonio Fraga Mouret" La Raza National Medical Center of the Mexican Institute of Social Security (IMSS), Mexico City, MEX; 2 Endocrinology and Thyroidology, General Hospital of Mexico "Dr. Eduardo Liceaga", Mexico City, MEX; 3 Surgery, Specialties Hospital "Dr. Antonio Fraga Mouret" La Raza National Medical Center of the Mexican Institute of Social Security (IMSS), Mexico City, MEX; 4 Internal Medicine, General Hospital, La Raza National Medical Center of the Mexican Institute of Social Security (IMSS), Mexico City, MEX

**Keywords:** cervical lymph node metastasis, ectopic ptc, ectopic thyroid, papillary thyroid carcinoma, thyroid hyperplasia, ultrasound

## Abstract

Cervical lymph nodes can indicate various inflammatory or cancerous conditions. Ectopic thyroid carcinoma, particularly papillary carcinoma, can be found in resected lymph nodes even without clinical thyroid abnormalities, known as hidden (incidental) metastasis. We present the case of a 42-year-old woman with a 5 cm thyroid nodule and associated symptoms. Ultrasound and biopsy confirmed a multinodular goiter and an incidental metastatic papillary thyroid carcinoma (PTC) in the right cervical lymph node. A total thyroidectomy and lymph node exploration revealed PTC metastasis. Treatment included 100 mCi ablation, resulting in a complete response. This case emphasizes the need for careful evaluation and precise treatment planning for patients with PTC to avoid misdiagnosis.

## Introduction

Various inflammatory or cancerous conditions are associated with signs of cervical lymph nodes. Further research has revealed histopathological signs of abnormal thyroid tissue in cervical lymph nodes, identified through epithelial protein markers, such as keratin, which are found in dissected lymph nodes of patients with other cancerous conditions [1,2]. Other studies have found thyroid cancer tissue in cervical nodules incidentally during elective resection and surgical treatments for patients with squamous cell carcinoma of the head and neck [3]. Specifically, they have found ectopic papillary thyroid cancer in cervical nodes without any abnormalities or primary carcinoma present in the thyroid gland, which is referred to as occult (incidental) cancer [4].

Papillary thyroid carcinoma originates in the tissue of the cervical lymph nodes, indicating that thyroid cancer can spread to these nodes. Ectopic thyroid tissue can undergo the same pathological processes as normal thyroid tissue, including inflammation, hyperplasia, and tumor formation. Differentiating between primary cancer in ectopic thyroid tissue and cancer that has spread to cervical lymph nodes is crucial in managing patients with a history of thyroid cancer. As a result, the preferred treatment for papillary thyroid carcinomas has been the surgical removal of the thyroid gland and cervical lymph nodes [5].

## Case presentation

A 42-year-old woman was referred for evaluation of a large thyroid nodule. She experienced symptoms such as weakness, fatigue, difficulty swallowing, and changes in her voice but had not lost weight.

**Figure 1:**
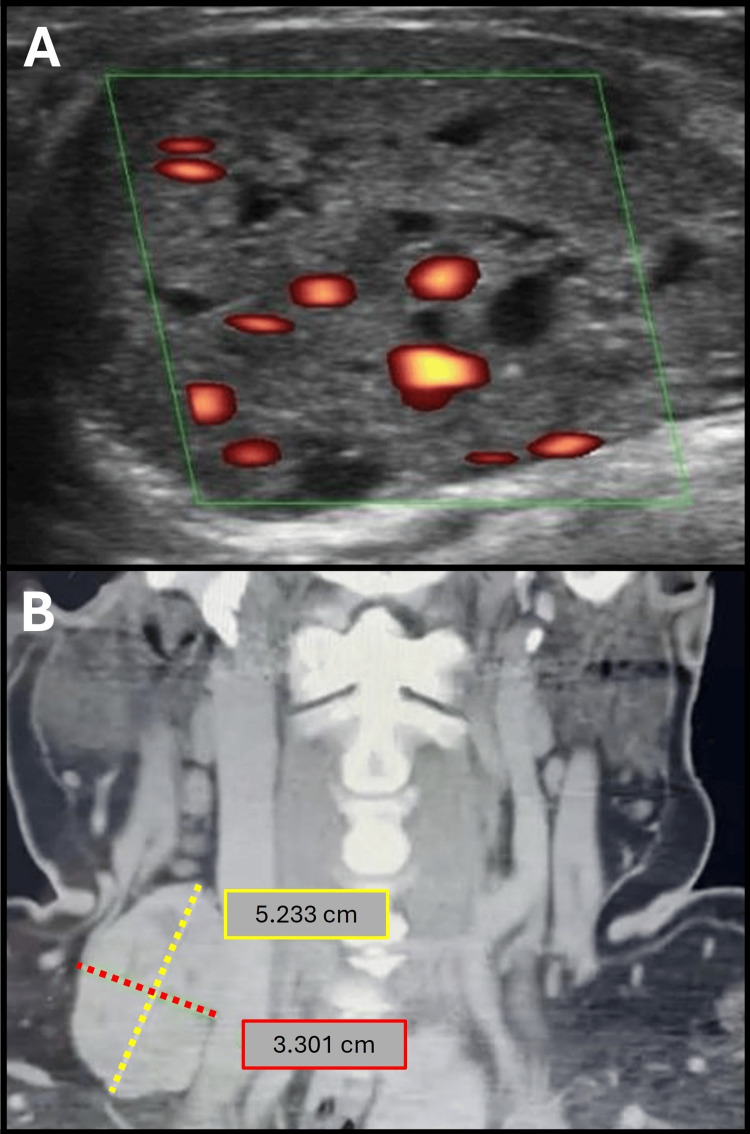
Preoperative Doppler ultrasonography and CT scan (A) Mild vascular patterns of the thyroid nodules appear on color Doppler ultrasound. (B) The coronal view shows an encapsulated mass in the right cervical lymph node, measuring 5.23 cm in length and 3.30 cm in width, with heterogeneous contrast enhancement.

An ultrasound revealed a grade III multinodular goiter with signs of possible extension into the chest. Additionally, a swollen lymph node measuring 5.23 cm in length and 3.30 cm in width was found on the right side of her neck on the computed tomography angiography (CTA) (Figure 1).

**Figure 2:**
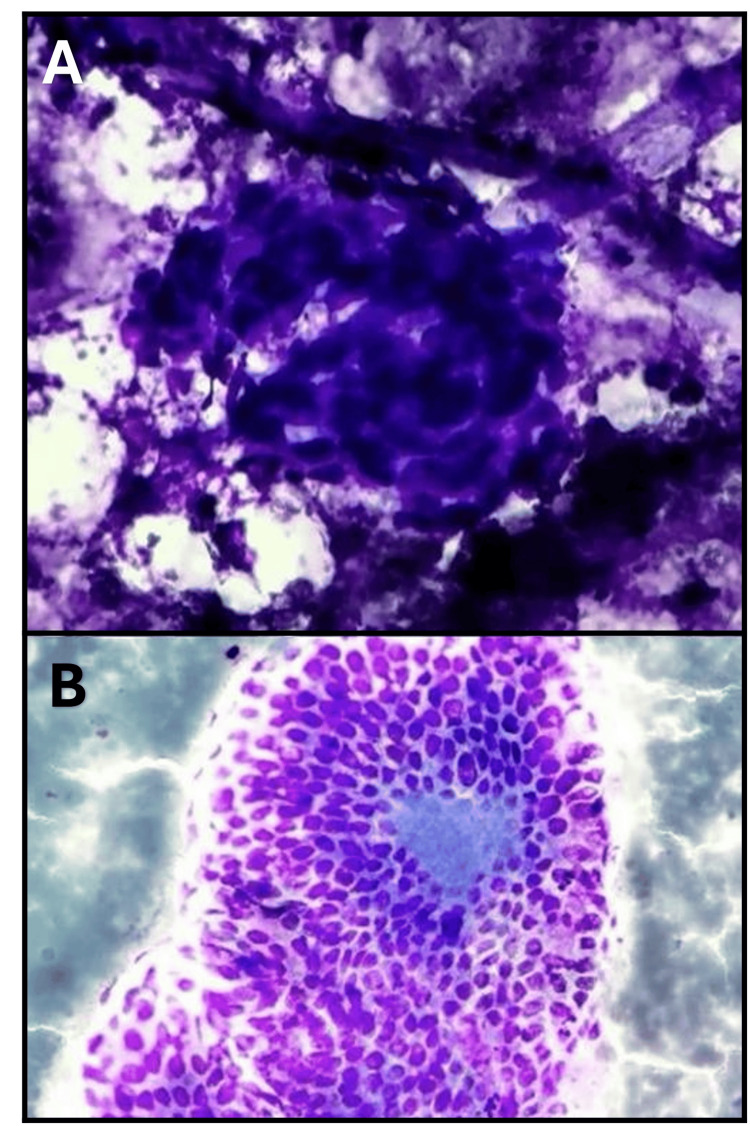
FNAB of thyroid and the lymph node (A) Cytology of fine needle aspiration biopsy of a cervical lymph node (hematoxylin-eosin stain, × 400). (B) Pathologic examination confirmed the diagnosis of an ectopic papillary thyroid carcinoma (periodic acid-schiff stain, × 100). FNAB: fine-needle aspiration biopsy

A fine-needle aspiration biopsy (FNAB) was done on the thyroid and lymph nodes. The results indicated that the thyroid nodule was benign (Bethesda II) while the biopsy of the right cervical lymph node showed the presence of metastatic papillary thyroid carcinoma (Figure 2).

The patient underwent a complete thyroidectomy and lymph node exploration, as well as the removal of a cervical lesion that showed an ectopic papillary thyroid carcinoma. The examination results showed nodular thyroid hyperplasia with two adenomatoid nodules measuring 3.5 cm in length and 1.5 cm in width on the right lobe. No carcinoma was found in the thyroid tissue. Additionally, there was evidence of ectopic papillary thyroid carcinoma in the fatty tissue near the thyroid isthmus, measuring 0.4 cm. Cervical lymph node dissection on the left side revealed affected lymph nodes with classic pattern papillary thyroid carcinoma, measuring 5.5 cm in the major axis without any capsule rupture. A body scan showed increased uptake in the surgical area (Figure 2). A 100 mCi ablation treatment was administered, and subsequent evaluations indicated a complete structural and biochemical response.

## Discussion

Cervical lymph nodes can be affected by both inflammatory and neoplastic conditions and may become enlarged in malignant tumors. Asymptomatic ectopic thyroid tissue can be present in these lymph nodes and is sometimes incidentally found during treatment for head and neck carcinomas. It can appear as either an asymptomatic tumor in the neck or as hyperfunctioning ectopic thyroid tissue [6,7].

Thyroid malignancy can occur in 3% of patients with ectopic thyroid tissue, mainly involving papillary carcinomas. It can be challenging to determine whether the cancer is metastatic thyroid carcinoma or ectopic thyroid carcinoma [8]. There have been reports that thyroid and salivary carcinomas can develop from ectopic tissues. Attie JN et al. reported that thyroid gland carcinoma could be found in patients with thyroid carcinoma in the cervical lymph node [9]. They conducted a histological examination of clinically normal thyroids in patients with occult metastatic thyroid carcinoma, revealing thyroid cancer in all patients who underwent thyroidectomy. Pacheco-Ojeda L et al. also reported six suspicious cases of occult metastasis and found thyroid gland carcinomas in five of these cases [10].

The frequency of occult thyroid gland carcinoma varies significantly, according to reports. Extensive studies, such as those by Yamamoto et. al., indicate that incidental thyroid carcinoma of the lymph nodes does not necessarily seem to be associated with thyroid gland carcinoma, meaning it is not always metastatic [11]. The relatively high incidence of papillary carcinoma compared to its incidence in the thyroid gland suggests a non-metastatic origin of thyroid carcinoma in the lymph nodes. If the thyroid carcinomas were metastatic, the "primary carcinoma" of the thyroid gland should naturally grow, even if the growth is very slow [12].

The removal of ectopic thyroid tissue was previously recommended to eliminate metastatic foci from thyroid cancer. However, recent research has shown that over 70% of ectopic thyroid cases are benign. Therefore, it is essential to avoid unnecessary removal of ectopic thyroid tissue, as patients with lingual thyroid may lack up to 75% of normal thyroid tissue, potentially leading to consequences such as hypothyroidism [13]. Caution should be exercised when performing a total thyroidectomy without removing the lateral ectopic thyroid, as this may reduce the effectiveness of radioiodine therapy. However, patients who have received radioactive iodine therapy should still have their ectopic thyroid tissue removed. This should be done carefully, and ectopic tissue should only be removed in areas where normal thyroid anatomy is absent after confirming detectable malignancy [14,15].

## Conclusions

We report a case of a patient with a neck tumor displaying diverse symptoms, which prompted a biopsy. The biopsy confirmed the presence of papillary thyroid cancer outside the thyroid gland. Interestingly, the initial fine-needle aspiration biopsy of the thyroid gland indicated thyroid hyperplasia. This unique case underscores the importance of identifying ectopic thyroid tissue and its potential for cancer. Timely detection and treatment can significantly impact the patient's prognosis and survival.
